# Effective osteoporosis treatment with teriparatide is associated with enhanced quality of life in postmenopausal women with osteoporosis: the European Forsteo Observational Study

**DOI:** 10.1186/1471-2474-14-251

**Published:** 2013-08-22

**Authors:** Östen Ljunggren, Annabel Barrett, Ivaylo Stoykov, Bente L Langdahl, Willem F Lems, J Bernard Walsh, Astrid Fahrleitner-Pammer, Gerald Rajzbaum, Franz Jakob, Dimitrios Karras, Fernando Marin

**Affiliations:** 1 Division of Endocrinology, Department of Medical Sciences, Uppsala University Hospital, Uppsala S-751 85, Sweden; 2 Lilly Research Centre, Windlesham, UK; 3 Aarhus University Hospital, Aarhus, Denmark; 4 VU University Medical Centre, Amsterdam, The Netherlands; 5 Trinity College, Dublin, Ireland; 6 Medical University, Graz, Austria; 7 Hôpital St. Joseph, Paris, France; 8 Julius-Maximilians-Universitaet, Wuerzburg, Germany; 9 Veterans Administration Hospital, Athens, Greece

**Keywords:** EQ-5D, Fracture, Osteoporosis, Quality of life, Teriparatide

## Abstract

**Background:**

To describe changes in health-related quality of life (HRQoL) of postmenopausal women with osteoporosis treated with teriparatide for up to 18 months and followed-up for a further 18 months, and to assess the influence of recent prior and incident fractures.

**Methods:**

The European Forsteo Observational Study (EFOS) is an observational, prospective, multinational study measuring HRQoL using the EQ-5D. The primary objective was to assess changes in HRQoL during 36 months in the whole study population. A secondary *post-hoc* analysis examined fracture impact on HRQoL in four subgroups classified based on recent prior fracture 12 months before baseline and incident clinical fractures during the study. Changes from baseline were analysed using a repeated measures model.

**Results:**

Of the 1581 patients, 48.4% had a recent prior fracture and 15.6% of these patients had an incident fracture during follow-up. 10.9% of the 816 patients with no recent prior fracture had an incident fracture. Baseline mean EQ-VAS scores were similar across the subgroups. In the total study cohort (n = 1581), HRQoL (EQ-VAS and EQ-5D index scores) improved significantly from baseline to 18 months and this improvement was maintained over the 18-month post-teriparatide period. Improvements were seen across all five EQ-5D domains during teriparatide treatment that were maintained after teriparatide was discontinued. Subjects with incident clinical fractures had significantly less improvement in EQ-VAS than those without incident fractures. Recent prior fracture did not influence the change in EQ-VAS during treatment.

**Conclusions:**

EFOS is the first longitudinal study in women with severe postmenopausal osteoporosis in the real world setting to show a substantial improvement in HRQoL during teriparatide treatment that was sustained during subsequent treatment with other medications. The increase in HRQoL was lower in the subgroups with incident fracture but was not influenced by recent prior fracture. The results should be interpreted in the context of the design of an observational study.

## Background

The quality of life of postmenopausal women with osteoporosis is adversely affected if they have bone fractures and pain [1-6]. Health-related quality of life (HRQoL) has become an important outcome measure in osteoporosis clinical trials and covers physical, emotional and social functioning and well-being, which can be assessed using generic or osteoporosis-specific questionnaires [1]. A decreased HRQoL is well documented in postmenopausal women with osteoporosis-related fractures, and this can vary according to type of fracture [7-9], number and severity of fractures [4,10,11], and time since fracture [12]. Some aspects of HRQoL are reduced in patients with subclinical vertebral fractures or reduced bone mineral density [13], although low BMD is generally regarded as asymptomatic. Additional factors, such as comorbidities and back pain, may also influence HRQoL, especially in older women [14,15].

Osteoporosis treatment aims to prevent fractures and, in turn, to reduce morbidity and mortality. The efficacy of osteoporosis medications in reducing the risk of fragility fractures in postmenopausal women with osteoporosis has been demonstrated in randomised controlled trials (RCTs), but there is only limited data that they are capable of improving patient quality of life [16-20] or reduce mortality [21]. Teriparatide is a bone anabolic agent that reduces the risk of vertebral and non-vertebral fractures in postmenopausal women [22], but its effect on patient quality of life has not been thoroughly investigated. Moreover, RCTs are conducted using carefully selected patients and there is limited data on HRQoL in routine clinical practice, where patients with osteoporosis can have multiple comorbidities, more severe disease, and receive sequential treatment regimens. In addition, patient adherence or persistence with anti-osteoporosis therapies is poor in everyday clinical practice [23,24] and may lead to an increased fracture risk and reduced quality of life. Therefore, data from observational studies can complement RCTs [25].

Results from the ICARO observational study showed that osteoporotic women who sustained a new fragility fracture during antiresorptive therapy had a lower HRQoL score compared with patients without an incident fracture [26]. Similarly, in the Observational Study of Severe Osteoporosis (OSSO), women with an inadequate response to osteoporosis therapy had a high rate of incident fractures during 12 months’ treatment with any osteoporosis medication, and this was associated with worse HRQoL regardless of prior fracture status [2]. An improvement in HRQoL during 18 months of teriparatide treatment in the European Forsteo Observational Study (EFOS) has been reported [27]. However, changes in HRQoL both during and after teriparatide treatment in routine clinical practice have not been described, nor has the influence of prior and incident fractures on HRQoL during such treatment.

EFOS was a large 36-month, prospective, observational study designed to evaluate fracture outcomes, back pain and HRQoL in postmenopausal women with severe osteoporosis treated with teriparatide in the outpatient setting for a maximum of 18 months, followed by a post-teriparatide treatment observational period of a further 18 months. A reduced incidence of clinical vertebral and non-vertebral fractures and reduction in back pain over 36 months has been reported elsewhere [28].

The primary aim of this paper is to present a pre-planned analysis of the HRQoL results in the EFOS study population, both during teriparatide treatment for up to 18 months and in the subsequent 18-month period after teriparatide discontinuation when patients were receiving other osteoporosis medications. In addition, we perform a secondary *post-hoc* analysis to examine the HRQoL changes in patients grouped according to recent prior fracture in the 12 months before the baseline assessment and incident clinical fracture during 36 months follow-up.

## Methods

### Study design and patients

EFOS was a multicentre, prospective, observational study of fracture outcomes, back pain and HRQoL in postmenopausal women with osteoporosis in eight European countries (Austria, Denmark, France, Germany, Greece, Ireland, the Netherlands, Sweden); the study design, sample size considerations, and characteristics of the patient population have been described in detail elsewhere [29]. Briefly, 1649 postmenopausal women with a diagnosis of osteoporosis who were about to initiate teriparatide treatment were enrolled. Patients were followed for the duration of their teriparatide treatment, which they could discontinue at any time, and were asked to return for two additional visits after they discontinued teriparatide. Patients were excluded from the study if they were currently being treated with an investigational drug or procedure, or had any contraindications as described in the teriparatide label. The observational study design meant there were no further restrictions for the selection of patients, reflecting routine practice. All patients gave written informed consent prior to enrolment and were able to withdraw without consequence at any time. The study was approved by local ethics committees or review boards, depending on local requirements. The study was conducted from April 2004 (first patient enrolled) until February 2009 (last patient completed).

### Data collection

At the baseline visit, patient demographic characteristics, risk factors for osteoporosis and falls, osteoporosis therapies and disease status were recorded [29]. The women attended follow-up visits at approximately 3, 6, 12, and 18 months after teriparatide initiation, and at 6 and 18 months after discontinuing teriparatide treatment, during which time the majority of patients took other osteoporosis medication, mainly bisphosphonates [27].

HRQoL was measured at each visit using the EQ-5D, a generic self-administered health status questionnaire that consists of two parts [30]. In the first part, patients classify their own health status according to five dimensions of health (mobility, self-care, usual activities, pain/discomfort, and anxiety/depression) each of which is scored on a three-point scale (no problems, some problems, or extreme problems). From the scores of these five dimensions, a single index value is derived using a general UK population-based algorithm, where an index score of 0 represents a state equivalent to death and a score of 1 represents a state of perfect health [31,32]. In the second part of the EQ-5D, patients complete a visual analogue scale (EQ-VAS), which assesses their perceived overall health status on the day of scoring on a scale from 0 (worst imaginable health state) to 100 (best imaginable health state).

At the baseline assessment, a patient’s history of fragility fractures since the age of 40 years was collected by recording the fracture location and date of each fracture. From this information, a history of recent prior fracture within the last 12 months before starting teriparatide was determined and categorised as yes or no. Information on incident clinical vertebral and non-vertebral fragility fractures occurring during the study were collected at each follow-up visit as self-reported by the patient; these fractures were then diagnosed and confirmed by review of the original radiographs and/or the radiology or surgical reports at the investigational site. A new or worsened vertebral fracture was defined from the presence of a confirmed radiographic vertebral fracture associated with signs and/or symptoms suggestive of a vertebral fracture (such as acute or severe back pain). Incident morphometric spine fractures were not analysed in EFOS.

### Statistical analysis

Data were analysed for the total study cohort, which included all patients with a baseline visit and at least one follow-up visit. The impact of fractures on patient HRQoL was assessed in a post-hoc analysis by classifying the total study population into four mutually exclusive subgroups based on recent prior fracture in the 12 months before baseline (yes/no) and incident fracture during the study (yes/no).

Descriptive statistics (e.g. frequencies and percentages for categorical variables; means and standard deviations (SD) or medians with 25^th^ and 75^th^ percentiles for continuous variables) were used to describe the total study population and subgroups by recent prior and incident fracture status. For EQ-VAS and EQ-5D index scores, a last observation carried forward (LOCF) approach was used for missing data.

Baseline characteristics between subgroups were compared using Fisher exact tests for categorical variables, Wilcoxon rank sum tests for time since most recent fracture and number of previous fractures, and *t*-tests for other continuous variables. The *p*-values of the pairwise comparisons were Bonferroni adjusted.

EQ-VAS changes from baseline were analysed using a mixed model for repeated measures (MMRM) with an unstructured correlation matrix and adjusting for age, duration of prior bisphosphonate treatment and a diagnosis of rheumatoid arthritis at baseline. We adjusted for age because it is associated with HRQoL [31]. Likewise, we adjusted for rheumatoid arthritis because it was the most prevalent comorbidity in the patient cohort and is associated with worse HRQoL regardless of fracture status. Duration of prior bisphosphonate use was included in the model as a surrogate of the severity of osteoporosis and for consistency with the model used in the primary analysis of the study (incident fractures). Missing values are taken into account using this method. The data are presented as adjusted mean changes from baseline obtained after controlling for the covariates (least-squares mean changes) with standard errors. *P*-values for the within-group change from baseline represent the unique influence of the corresponding factor after adjustment for the other factors in the model. Pairwise comparisons between subgroups (by fracture status) in the adjusted least-squares mean change from baseline at each follow-up visit were calculated, and the *p*-values for the comparisons are presented.

For the EQ-5D index score, which was not normally distributed, Wilcoxon signed-rank test was used to assess within-group changes from baseline. Between-group comparisons used Wilcoxon rank-sum tests and Bonferroni adjusted *p*-values within each visit are reported.

All p-values presented are two-sided and the level of significance is set to 5%. All data were analysed using SAS software version 9.2.

## Results

### Patients

**Figure 1:**
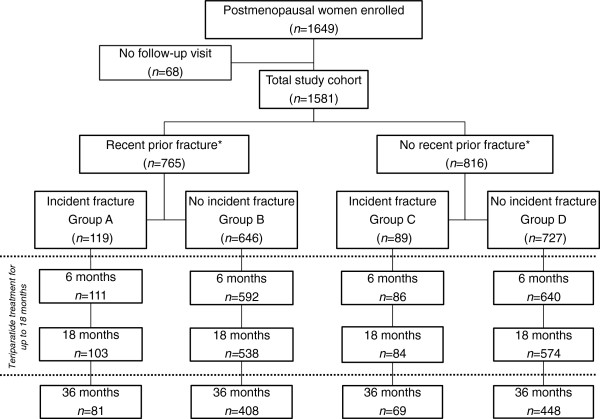
**Study disposition.** Patient distribution by recent prior fracture in the 12 months before study entry and by incident fracture during the study.

Of the 1649 postmenopausal women enrolled in the study, 1581 were analysed at baseline and returned for at least one post-baseline visit (the total study cohort). Figure 1 presents the patient distribution for the secondary analysis according to recent prior fracture status in the 12 months before study entry and incident clinical fracture during the study. Of the 1581 patients, 765 (48.4%) had a recent prior fracture in the 12 months before study entry. Of these, 119 (15.6%) patients had an incident fracture during the study. In the group of patients with no prior fracture in the 12 months before baseline (*n* = 816), 89 (10.9%) patients had an incident fracture during the study. Across the subgroups, 79–94% of patients remained in the study at 18 months and 62–78% were observed at the 36-month visit.

Table 1 summarises the baseline characteristics of the total study cohort and the four subgroups by recent prior and incident fracture status. There were few differences in baseline characteristics between the subgroups. As expected (because of how the subgroups were defined), the time since recent fracture and number of previous fractures after age 40 differed between groups. However, for the time since most recent fracture, no difference was detected between patients with and without incident fractures in the subgroups with recent prior fracture in the 12 months before baseline (groups A and B); the same was true for the subgroups without recent prior fracture (groups C and D). Among the patients with recent prior fracture, those with an incident fracture (group A) had a significantly higher number of previous fractures than those without incident fracture (group B, *p* < 0.05); no difference was seen between patients with and without incident fracture in the groups with no recent prior fracture (groups C and D).

**Table 1:** Baseline characteristics of the four subgroups and total study cohort

	**Total study cohort (*n*** **= 1581)**	**Group A**	**Group B**	**Group C**	**Group D**
		**Recent prior fracture**^**a**^**; incident fracture (*n*** **= 119)**	**Recent prior fracture**^**a**^**; no incident fracture (*n*** **= 646)**	**No recent prior fracture**^**a**^**; incident fracture (*n*** **= 89)**	**No recent prior fracture**^**a**^**; no incident fracture (*n*** **= 727)**
Age (years)	71.0 (8.4)	72.1 (8.6)	71.3 (8.1)	70.6 (9.0)	70.5 (8.5)
Caucasian, %	99.2	100	99.1	100	99.1
Body Mass Index (kg/m^2^)	25.1 (4.3)	25.1 (4.6)	25.3 (4.2)	24.3 (4.0)	25.1 (4.3)
Early menopause (<40 years of age), %	8.9	7.9	7.9	11.9	9.6
Surgical menopause, %	18.1	20.2	15.9	23.6	19.0
Nulliparous, %	13.0	11.8	12.8	11.5	13.6
Current smoker, %	13.0	10.9	10.9	19.5	14.5
Osteoporotic hip fracture in mother, %	20.8	22.3	22.2	18.2	19.7
Time since most recent fracture (years)	2.0 (3.4)	0.3 (0.3)	0.3 (0.3)	4.5 (3.9)**	4.2 (4.1)**
Number of previous fractures after 40 years of age	2.9 (2.0)	**4.0 (2.0)#**	3.3 (1.9)	3.3 (2.3)	**2.3 (1.9)††**
Lumbar spine BMD T-score	−3.26 (1.16)	−3.26 (1.16)	−3.18 (1.22)	−3.45 (1.02)	−3.31 (1.13)
Total hip BMD T-score	−2.61 (1.05)	−2.82 (0.87)	−2.53 (1.11)	−2.79 (1.12)	−2.61 (1.00)
Back pain VAS	57.8 (26.6)	60.1 (26.5)	58.8 (26.2)	60.0 (25.2)	56.1 (27.1)
Uses arms when stands up from chair, %	63.3	70.6	65.0	47.7*‡	62.6
Sight problems, %	45.0	54.6	43.2	52.3	44.1
>1 fall in last year, %	42.0	53.8	45.0	42.0	37.4*
Prior osteoporosis medication, %	91.8	96.6	89.6	**98.9‡**	92.2
Prior bisphosphonate medication, %	73.4	76.5	70.7	**87.6‡#**	73.6
Comorbidities, %					
Rheumatoid arthritis	11.9	15.1	**13.9‡**	16.9	8.9
Chronic obstructive pulmonary disease	8.7	13.4	9.0	5.6	8.1
Diabetes mellitus	5.5	6.7	5.1	4.5	5.8
Concomitant medications, %					
Antihypertensives	37.2	37.8	38.8	31.4	36.4
Glucocorticoids	14.8	20.2	15.4	19.8	12.8
Thyroid hormones	13.3	10.9	15.1	18.6	11.6
Benzodiazepines	12.0	16.0	11.5	16.3	11.3
Antidepressants	10.2	**17.6‡**	10.6	12.8	8.4

Data are presented as mean (SD) unless indicated otherwise.

^a^Recent prior fracture within the last 12 months before baseline visit.

Comparisons between groups used Fisher exact tests for categorical variables, Wilcoxon signed-rank tests for time since most recent fracture and number of previous fractures, and *t*-tests for other continuous variables (all Bonferroni adjusted *p*-values).

*p < 0.05, **p < 0.001 for pairwise comparisons with groups A and B.

††*p* < 0.001 for pairwise comparisons with groups A, B, and C.

‡ *p* < 0.05 for pairwise comparison with group D.

# *p* < 0.05 for pairwise comparison with group B.

### EQ-VAS

**Figure 2:**
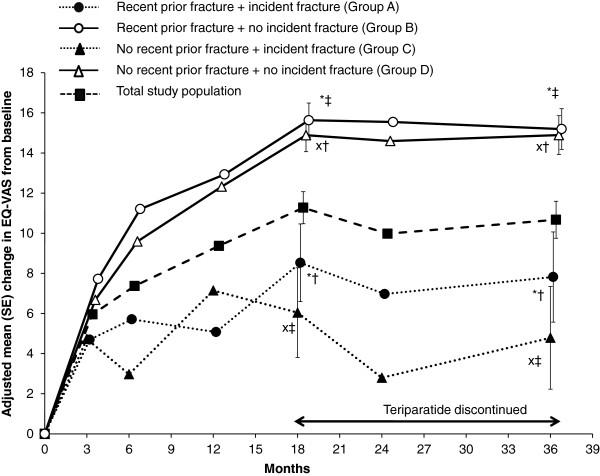
**EQ-VAS changes from baseline.** Adjusted mean (SE) change in EQ-VAS from baseline during and after teriparatide treatment in the total study cohort and according to recent prior fracture (within last 12 months) and incident fracture status. Data presented is from the MMRM analysis (adjusted for age, duration of prior bisphosphonate treatment and a diagnosis of rheumatoid arthritis at baseline). All *p* < 0.05 for within-group change from baseline (except for no recent prior/incident fracture at 6, 24 and 36 months). Significant pairwise comparisons between subgroups at 18 and 36 months are shown in the figure but the same pairwise comparisons were significant (*p* < 0.05) at 6, 12 and 24 months. *p < 0.05 for recent prior/incident fracture (group A) versus recent prior/no incident fracture (group B); †*p* < 0.05 for recent prior/incident fracture (group A) versus no recent prior/no incident fracture (group D); ‡ *p* < 0.001 for recent prior/no incident fracture (group B) versus no recent prior/incident fracture (group C); X *p* < 0.001 for no recent prior/incident fracture (group C) versus no recent prior/no incident fracture (group D). Note: variability (standard error bars) only added to the graph at two time points (18 and 36 months) for clarity.

Table 2 presents the unadjusted EQ-VAS scores at each visit for the total study population and for the four subgroups by recent prior and incident fracture status. These unadjusted results are derived from the available data at the visits and support the adjusted analysis from the MMRM (adjusted for age, duration of prior bisphosphonate treatment and a diagnosis of rheumatoid arthritis) shown in Figure 2. For the total study cohort, the unadjusted mean EQ-VAS score was 52.0 (SD 22.0) at baseline, increasing to 67.5 (SD 21.4) at 18 months and maintained at this level at 24 and 36 months (Table 2). The within-group change from baseline was significant at all visits (*p* < 0.001, Wilcoxon signed rank test). Figure 2 shows an improvement in adjusted mean EQ-VAS from baseline at all visits in the total study cohort (*p* < 0.001 from MMRM); the increase from baseline at 18 months was 11.3 (95%CI 9.7–12.8) and this was maintained during the post-teriparatide treatment period: 10.0 (95%CI 8.3–11.7) at 24 months and 10.7 (95%CI 8.9–12.5) at 36 months.

**Table 2:** Unadjusted EQ-VAS and EQ-5D index scores at each study visit

**Visit**	**Total study population (*N*** **= 1561)**		**Group A**		**Group B**		**Group C**		**Group D**	
			**Recent prior fracture**^**a**^**; incident fracture (*N*** **= 119)**		**Recent prior fracture**^**a**^**; no incident fracture (*N*** **= 646)**		**No recent prior fracture**^**a**^**; incident fracture (*N*** **= 89)**		**No recent prior fracture**^**a**^**; no incident fracture (*N*** **= 727)**	
	***n***		***n***		***n***		***n***		***n***	
***EQ-VAS, mean (SD)***										
Baseline	1558	52.01(22.0)	117	48.4 (22.0)	636	51.4 (21.8)	88	49.3 (21.6)	717	53.5 (22.1)
3 months	1434	59.0 (19.8)	105	54.9 (18.9)	584	59.3 (19.6)	81	55.2 (17.4)	664	59.8 (20.4)
6 months	1401	61.94(20.1)	110	56.1 (17.8)*†	579	63.3 (19.0)	85	53.6 (19.8)‡	627	62.9 (21.0)
12 months	1304	64.5 (21.4)	106	56.5 (20.7)**††	528	65.5 (20.5)	84	57.9 (17.9)‡	586	66.0 (22.4)
18 months	1249	67.5 (21.4)	101	60.6 (20.9)**††	514	68.8 (20.5)	79	57.8 (18.8)‡‡	555	68.9 (22.1)
24 months	1068	67.4 (22.4)	91	58.9 (24.1)**†	416	69.6 (20.9)	71	54.8 (21.0)‡‡	490	68.9 (22.6)
36 months	951	68.7 (22.5)	79	60.1 (23.7)*†	385	70.2 (21.6)	65	57.9 (22.4)‡‡	422	70.7 (22.3)
Endpoint^b^	1513	64.6 (23.1)	116	57.1 (24.3)*†	615	65.5 (22.5)	84	55.7 (22.5)‡‡	698	66.2 (23.2)
***EQ-5D index score, median (Q1, Q3)***										
Baseline	1535	0.59 (0.08,0.73)	116	0.23 (−0.02, 0.67)††	623	0.59 (0.03, 0.73)	86	0.57 (0.17, 0.69)	710	0.59 (0.09, 0.73)
3 months	1409	0.69 (0.52, 0.76)	103	0.62 (0.29, 0.73)*†	577	0.69 (0.52, 0.76)	80	0.62 (0.23, 0.74)	649	0.69 (0.52, 0.80)
6 months	1367	0.69 (0.52, 0.80)	108	0.62 (0.52, 0.71)**††	564	0.69 (0.59, 0.80)	83	0.62 (0.26, 0.73)‡‡	612	0.69 (0.59, 0.80)
12 months	1289	0.69 (0.59, 0.80)	106	0.69 (0.52, 0.76)*††	522	0.69 (0.59, 0.80)	85	0.62 (0.26, 0.73)‡‡	576	0.73 (0.59, 0.85)
18 months	1233	0.73 (0.59, 0.85)	102	0.66 (0.52, 0.74)**††	513	0.73 (0.62, 0.85)	80	0.62 (0.52, 0.76)‡‡	538	0.73 (0.59, 0.85)
24 months	1057	0.73 (0.59, 0.85)	87	0.62 (0.09, 0.73)**††	410	0.73 (0.62, 0.88)	73	0.69 (0.31, 0.80)‡	487	0.73 (0.59, 0.88)
36 months	939	0.73 (0.59, 0.88)	75	0.62 (0.15, 0.76)**††	377	0.76 (0.62, 1.00)	66	0.69 (0.52, 0.80)‡	421	0.76 (0.62, 1.00)
Endpoint^b^	1483	0.69 (0.52, 0.80)	111	0.59 (0.09, 0.76)**††	605	0.69 (0.59, 0.80)	85	0.69 (0.36, 0.76)‡	682	0.73 (0.52, 0.85)

For the EQ-VAS and EQ-5D index scores, a higher score represents a better quality of life.

Teriparatide treatment was initiated at baseline and continued for up to 18 months; other osteoporosis medications were used between 18 and 36 months after teriparatide was discontinued.

^a^Recent prior fracture within the last 12 months before baseline visit.

^b^Missing data was handled using the last observation carried forward (LOCF) method.

Between-group comparisons (Wilcoxon rank-sum test; Bonferroni adjusted *p*-values).

*p < 0.05, ** *p* < 0.001 for pairwise comparison of group A versus group B.

†*p* < 0.05, ††*p* < 0.001 for pairwise comparison of group A versus group D.

‡*p* < 0.05, ‡‡*p* < 0.001 for pairwise comparison of group C versus groups B and D.

In the secondary *post-hoc* analysis, the four subgroups of patients according to recent prior and incident fracture status did not differ in their baseline mean EQ-VAS scores (Table 2). Figure 2 shows significant within-group changes from baseline in the adjusted mean EQ-VAS for all subgroups (*p* < 0.05 from MMRM), except for the no recent prior/incident fracture subgroup at 6, 24 and 36 months. Figure 2 also shows that the two subgroups with incident fracture (dotted lines) had significantly less improvement in adjusted mean EQ-VAS (irrespective of recent prior fracture) than the two subgroups without incident fracture (solid lines, *p* < 0.05 for all comparisons at 18 and 36 months). Notably, the no recent prior fracture plus no incident fracture subgroup showed a clear increase in EQ-VAS from baseline to 18 months that was maintained at 36 months (as did the recent fracture plus no incident fracture subgroup). Pairwise comparisons revealed no significant differences at any visit in the change in EQ-VAS between the two subgroups with incident fractures (Groups A and C) or between the two subgroups with no incident fracture (Groups B and D) (Figure 2), indicating that recent prior fracture does not influence the change in HRQoL.

### EQ-5D index scores

Median (Q1, Q3) EQ-5D index scores for the total study cohort increased from 0.59 (0.08, 0.73) at baseline to 0.73 (0.59, 0.85) at 18 months and were maintained at this median value after teriparatide discontinuation (Table 2). The median (Q1, Q3) change from baseline in EQ-5D index scores for the total study cohort (Table 3) was significant at each follow-up visit (*p* < 0.001, Wilcoxon signed rank test for within-group change).

**Table 3:** Median change from baseline in EQ-5D index score

**Visit**	**Total study cohort (*n*** **= 1581)**	**Group A**	**Group B**	**Group C**	**Group D**
		**Recent prior fracture**^**a**^**; incident fracture (*n*** **= 119)**	**Recent prior fracture**^**a**^**; no incident fracture (*n*** **= 646)**	**No recent prior fracture**^**a**^**; incident fracture (*n*** **= 89)**	**No recent prior fracture**^**a**^**; no incident fracture (*n*** **= 727)**
3 months	0.07 (0.00, 0.37)	0.07 (0.00, 0.50)	0.10 (0.00, 0.51) ^‡^	0.04 (0.00, 0.27)	0.04 (0.00, 0.27)
6 months	0.11 (0.00, 0.46)	0.10 (0.00, 0.53)	0.14 (0.00, 0.53) ^†^	0.04 (0.00, 0.26)	0.11 (0.00, 0.41)
12 months	0.12 (0.00, 0.46)	0.17 (0.00, 0.60) ^*^	0.14 (0.00, 0.53) ^†^	0.04 (−0.05, 0.27) ^#^	0.11 (0.00, 0.43)
18 months	0.15 (0.00, 0.50)	0.17 (−0.03, 0.57)	0.20 (0.00, 0.53) ^†^	0.04 (−0.04, 0.40)	0.13 (0.00, 0.43)
24 months	0.17 (0.00, 0.47)	0.10 (−0.04, 0.50) ^§^	0.21 (0.00, 0.57) ^†, ‡^	0.11 (−0.07, 0.33)	0.11 (0.00, 0.38)
36 months	0.17 (0.00, 0.48)	0.15 (−0.07, 0.60)	0.20 (0.00, 0.57) ^†^	0.09 (0.00, 0.33)	0.16 (0.00, 0.38)

^a^Recent prior fracture within the last 12 months before baseline visit.

Data given in brackets are the interquartile range (Q1, Q3).

All *p* < 0.05 for within-group change from baseline (Wilcoxon signed rank test).

Between group comparisons at each visit (Wilcoxon rank-sum tests, Bonferroni adjusted *p*-values).

^*^p < 0.05 for pairwise comparison of group A versus group C.

^†^*p* < 0.05 for pairwise comparison of group B versus group C.

^‡^*p* < 0.05 for pairwise comparison of group B versus group D.

^#^*p* < 0.05 for pairwise comparison of group C versus group D.

^§^*p* < 0.05 for pairwise comparison of group A versus group B.

In the secondary *post-hoc* analysis, the baseline median EQ-5D index scores differed significantly between the subgroup with recent prior and incident fractures (group A) and the subgroup without recent prior and incident fractures (group D) (0.23 [Q1, Q3: -0.02, 0.67] vs. 0.59 [Q1, Q3: 0.09, 0.73]; *p* < 0.001); all other pairwise comparisons at baseline were non-significant (Table 2). The median (Q1, Q3) changes from baseline in EQ-5D index scores for the four subgroups by recent prior and incident fracture status (Table 3) shows large variability at each visit, but there was a trend for improvement to 18 months that was maintained to 36 months (*p* < 0.05 for within-group change from baseline for each subgroup, Wilcoxon signed rank test). Pairwise comparisons in the change from baseline at the 18 and 36 month visits showed there was significantly less improvement in EQ-5D index score in the subgroup with no recent prior/incident fracture (group C) compared with the recent prior/no incident fracture subgroup (group B) (Bonferroni adjusted *p* values; *p* < 0.05 at both time points); all other pairwise comparisons at these two time points were non-significant (Table 3).

### EQ-5D domains

**Figure 3:**
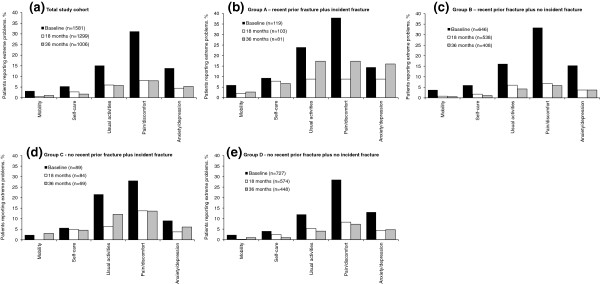
**Extreme problems in EQ-5D domains.** Patients (%) reporting extreme problems at baseline and at 18- and 36-months follow-up in the five domains of EQ-5D for **(a)** the total study cohort, **(b)** group A with recent prior fracture and incident fracture; **(c)** group B with recent prior fracture and no incident fracture; **(d)** group C with no recent prior fracture and incident fracture; and **(e)** group D with no recent prior fracture and no incident fracture. The remaining patients in each group reported either no problems or some problems for each domain.

For the total study cohort, there were improvements from baseline in all five EQ-5D domains during teriparatide treatment that were maintained after teriparatide was discontinued. The percentage of women reporting extreme problems in the five domains was reduced at all post-baseline visits and Figure 3 shows the results at 18 and 36 months. The highest frequency of extreme problems was in the usual activities and pain/discomfort domains.

Figure 3 also shows the percentage of women reporting extreme problems in the five EQ-5D domains at baseline and 18 and 36 months follow-up for the four subgroups according to recent prior and incident fracture status. As expected, for all five domains the frequency of extreme problems was higher at baseline for the two subgroups with recent prior fracture (groups A and B) than the two groups with no recent prior fracture (groups C and D). Also, the frequency of extreme problems was higher at 18- and 36-months follow-up in the subgroups with incident fractures (groups A and C) than in the subgroups with no incident fractures (groups B and D). Thus, the proportion with extreme problems was highest at baseline and during follow-up in the subgroup of women with both recent prior and incident fractures (group A). The proportion of patients reporting extreme problems in the two subgroups with no incident fractures (groups B and D) decreased during follow-up.

## Discussion

EFOS is the first study to longitudinally examine health-related quality of life in postmenopausal women with severe osteoporosis in routine clinical practice both during and after teriparatide treatment. The results show that HRQoL (measured using the EQ-5D) is substantially improved during teriparatide treatment for up to 18 months and, importantly, is maintained during the 18-month post-teriparatide period while patients are receiving other osteoporosis treatments (mainly bisphosphonates, as described previously by Fahrleitner-Pammer et al. [27]). Notably, improvements were seen across all five EQ-5D domains during teriparatide treatment. The HRQoL results are consistent with the back pain results already reported for this study cohort [27]. Our analysis also shows that when the quality of life data are broken down according to recent prior and incident clinical fracture status, all four subgroups have a comparable but low EQ-VAS at baseline and show a trend for improvement during the study. However, the increase in EQ-VAS is smaller in patients who had an incident fracture at some point during the study compared with those without an incident fracture. Recent prior fracture in the 12 months before the study does not appear to influence the change in EQ-VAS during the study.

Interestingly, the results for the subgroup with no recent prior fracture plus no incident clinical fracture showed a low level of HRQoL at baseline and an increase during treatment. Our finding of a low baseline HRQoL even in the subgroup with no recent prior fracture is consistent with a recent systematic review, which reported that HRQoL is adversely affected by osteoporosis in the absence of vertebral fracture [33]. However, it may also be a consequence of our patient stratification. We have previously reported that, at baseline, 91.9% of the women in the EFOS study had a previous fracture since the age of 40 years and 70% had two or more vertebral deformities [28]. As those with prior fractures older than 12 months at baseline were included in the no recent prior fracture group, the improved HRQoL in the no recent prior/no incident fracture subgroup may be due to teriparatide effects on these older fractures. Teriparatide effects on other factors may also be involved in driving the quality of life improvement. These include relief of back pain, which is a major contributor to disability and has a negative impact on HRQoL [15]. Also, only clinical fractures were considered in EFOS and, as it has been shown that only about 30% of vertebral fractures are clinically diagnosed [34], patients may have undiagnosed fractures that are impacting on their quality of life [1]. It is possible that improvements in microfractures are contributing to the improved HRQoL and the beneficial effects of teriparatide on back pain may be related to the prevention and healing of microfractures [35,36], but this remains to be proven. The reduced risk of back pain in teriparatide-treated patients has been associated with a reduction in the severity and number of new vertebral fractures [37]. However, the anti-pain effect of teriparatide cannot be fully explained by vertebral fracture reduction or accelerated fracture healing, although there are very few publications suggesting what the possible reasons could be. A potential CNS effect of teriparatide cannot be excluded, but has not been shown so far [36]. Another possible explanation for the improvement in HRQoL is that it is a placebo effect resulting from the regular contact with study investigators. Further work is needed to identify other possible factors associated with the observed improvement in HRQoL during and after teriparatide treatment.

Our results are consistent with a substudy from the placebo-controlled Fracture Prevention Trial (FPT) of teriparatide treatment in postmenopausal women with severe osteoporosis, which examined the associations between fractures and HRQoL measured using the Osteoporosis Assessment Questionnaire [10,38]. All women in the FPT had a prevalent vertebral fracture at baseline and were at risk of subsequent fractures. Those with more severe vertebral fractures at baseline had a lower baseline HRQoL [10]. Women with incident fractures (vertebral and non-vertebral) had a worse HRQoL (physical function, symptoms and emotional status dimensions) during the study than women without incident fractures, regardless of treatment group [38]. Another large RCT evaluating denosumab treatment also demonstrated that incident clinical fractures have an adverse impact on HRQoL in postmenopausal women with osteoporosis [39].

We measured HRQoL using the generic EQ-5D questionnaire rather than an osteoporosis-specific questionnaire. EQ-5D is a validated standardised instrument for the measurement of health status. A generic instrument has advantages because it enables comparison of health effects between diseases and is the instrument of choice in health technology assessment. Previous studies using EQ-5D have shown an impaired quality of life status after fracture together with gradual improvements over the subsequent year [40]. Thus, EQ-5D is sensitive to change in patients with osteoporosis-related fractures. Although there have been no reports of what constitutes a clinically-relevant change in EQ-VAS scores for patients with osteoporosis, studies in other diseases indicate that EQ-VAS is responsive to changes in health and that a mean change in the score of 10.9 to 12.1 is a meaningful difference for improved health [41,42]. A change of 0.03 in the EQ-5D index score is considered a minimum clinically important difference in patients with osteoporosis [43].

At baseline, the unadjusted mean EQ-VAS score of 52.0 for the total study cohort indicated that patients had worse quality of life compared with the German population norm for women aged 70–79 years old (mean 75.5) [31]. We compared our results with population norms for Germany because this country provided 25% of the patients taking part in EFOS [29]. The mean EQ-VAS values at 18 and 36 months (67.5 and 68.7, respectively) showed improvement in HRQoL. Although HRQoL decreases with age in the general population [31], the EQ-VAS scores of patients participating in the present study increased over time; however, EQ-VAS was still lower than in an age-matched general German female population. In agreement with Dhillon et al. [14], we found the most problematic EQ-5D domains were pain/discomfort and usual activities; these two domains were also the most improved during teriparatide treatment.

Our results of an improvement in HRQoL during teriparatide treatment are consistent with a small retrospective single centre study of 57 patients with osteoporosis, which showed that HRQoL (assessed using the mini-Osteoporosis Quality of Life Questionnaire) improved with teriparatide treatment in a clinical practice setting [44]. Also, the PROPOSE observational study has shown HRQoL improvements during treatment with rhPTH(1–84) [45]. Although several randomised clinical trials have shown that other osteoporosis treatments (alendronate, strontium ranelate and zoledronic acid) have some beneficial effects on HRQoL in postmenopausal women [16-20], it is difficult to make comparisons between studies because of differences in study design, patient populations and methods used. Nevertheless, despite receiving previous osteoporosis treatment(s) and having multiple comorbidities, the patients in EFOS experienced substantial improvements in HRQoL during teriparatide treatment. As they received other osteoporosis medications after discontinuing teriparatide, those medications may have contributed to the maintenance of HRQoL enhancement associated with teriparatide treatment.

One of the most interesting findings of the study is the influence of recent prior and incident fracture on HRQoL. We observed that women with an incident fracture had less improvement in EQ-VAS during the study than patients with no incident fracture. However, recent prior fracture in the 12 months before baseline did not seem to influence the change in HRQoL either during or after teriparatide treatment. These findings are consistent with the OSSO study, which assessed the impact of fracture on HRQoL (EQ-5D and QUALEFFO) at baseline and at 6 and 12 months of treatment with any osteoporosis medication [2]. As in our study, HRQoL was worse in patients with an incident fracture regardless of recent prior fracture status [2]. Recent prior fracture in the months before baseline was associated with an increased risk of incident fracture and worse HRQoL at baseline compared with no recent prior fracture [2]. In contrast, however, Dhillon et al. [14] found no significant difference in EQ-5D scores between patients with and without a history of prior fracture.

Although incident fracture is associated with worse HRQoL, we cannot assume a causal relationship. In our analysis of EQ-VAS we adjusted for patient age, duration of prior bisphosphonate treatment and a diagnosis of rheumatoid arthritis, but many other factors may influence HRQoL, including other comorbidities, back pain, and lifestyle [46].

Our study has several limitations. First, this is a non-comparative study without a control group, and the subgroups of patients with incident fractures were small. The difference in sample size between the subgroups may limit the results. Second, incident fractures were those that were clinically recognised and patients could have had morphometric spine fractures not detected clinically. Third, EQ-5D assesses health status on the day of measurement and there were significant time gaps between HRQoL measurements. Fourth, the EQ-5D results may be influenced by patient drop-outs during the study. For EQ-VAS, this problem was reduced by applying the MMRM. For the other EQ-5D variables (EQ-5D index scores and domain scores), the LOCF method may have overestimated the improvement over time. Finally, comorbidities can impact on patient quality of life; although the baseline data suggests that the subgroups were reasonably well balanced regarding comorbidities.

The strengths of our study include the large sample size with few eligibility restrictions, allowing recruitment of a diverse range of subjects, many of whom had comorbidities and were taking concomitant medications. Other strengths include the prospective assessment of HRQoL both during and after teriparatide discontinuation in the normal clinical practice setting, and adjustment in the analysis for factors that may influence HRQoL, such as age, duration of prior bisphosphonate treatment and a diagnosis of rheumatoid arthritis. It also shows a clinically meaningful improvement in a relevant, patient-related outcome that is consistent across all patient groups.

## Conclusions

In this large pragmatic study of postmenopausal women with severe osteoporosis, we have observed a clinically meaningful improvement in HRQoL during teriparatide treatment that is maintained after teriparatide is discontinued when most patients are receiving other osteoporosis medications. We have demonstrated that the improvement in HRQoL is unaffected by a previous history of a recent fracture in the last 12 months before starting treatment but, as expected, was attenuated by incident clinical fractures. Further studies will be needed to understand the underlying mechanisms; however, this study for the first time demonstrated that a clinically-relevant improvement in HRQoL was maintained after stopping teriparatide treatment in addition to previously demonstrated positive effects on clinical fracture. However, because of the open label and non-comparative nature of the present study, our findings need confirmation through a randomised, controlled, double-blind study.

## Abbreviations

BMD: Bone mineral density; CI: Confidence interval; CNS: Central nervous system; EFOS: European Forsteo Observational Study; FPT: Fracture Prevention Trial; EQ-5D: EuroQol 5-Dimensions questionnaire; EQ-VAS: EuroQol Visual Analogue Scale; HRQol: Health-related quality of life; LOCF: Last observation carried forward; MMRM: Mixed model for repeated measures; OSSO: Observational Study of Severe Osteoporosis; RCT: Randomised controlled trial; rhPTH: Recombinant human parathyroid hormone; SD: Standard deviation.

## Competing interests

*O Ljunggren* has received lecture fees from, participates as a clinical investigator and is on advisory boards for Lilly, Amgen, Astra Zeneca and Nycomed. *BL Langdahl* has participated on advisory boards for Eli Lilly and Company, MSD, Amgen, Nycomed and Novartis, has received research grants from Eli Lilly and Company, MSD, Amgen and Novartis, and serves on Speaker’s Bureaus with Eli Lilly and Company, MSD and Amgen. *WF Lems* has received fees for speaking/advisory boards from MSD, Warner Chilcott, Eli Lilly, Amgen and Servier. *JB Walsh* has received honoraria for lectures from Servier, Eli Lilly and Company, MSD and Amgen. *A Fahrleitner-Pammer* has received research grants from Amgen, Eli Lilly and Company, Nycomed, Roche and Servier, and has contributed to Speaker’s Bureaus for Amgen, Daiichi Sankyo, Eli Lilly and Company, Genzyme, GSK, MSD, Novartis, Nycomed, Roche, Sanofi-Aventis and Servier. *F Jakob* has received honoraria for lectures and advice from Lilly, Amgen, Novartis, MSD, Nycomed, Servier and Roche, has received unrestricted research grants from Novartis, and is involved in clinical studies related to osteoporosis drugs initiated by Lilly, Amgen, Servier and Novartis. *F Marin* is a full-time employee and stock holder of Eli Lilly and Company. *A Barrett* and *I Stoykov* are employees of Eli Lilly and Company. *D Karras* and *G Rajzbaum* have nothing to disclose.

## Authors’ contributions

OL, BL, WL, JW, AF, GR, FJ, DK and FM contributed to the study design, revised and approved the final version of the manuscript. AB, IS and FM contributed to the analysis plan, drafted the manuscript and read and approved the final version. All authors read and approved the final manuscript.

## Pre-publication history

The pre-publication history for this paper can be accessed here:

http://www.biomedcentral.com/1471-2474/14/251/prepub
